# Increased Metabolite Levels of Glycolysis and Pentose Phosphate Pathway in Rabbit Atherosclerotic Arteries and Hypoxic Macrophage

**DOI:** 10.1371/journal.pone.0086426

**Published:** 2014-01-23

**Authors:** Atsushi Yamashita, Yan Zhao, Yunosuke Matsuura, Kazuaki Yamasaki, Sayaka Moriguchi-Goto, Chihiro Sugita, Takashi Iwakiri, Nozomi Okuyama, Chihiro Koshimoto, Keiichi Kawai, Nagara Tamaki, Songji Zhao, Yuji Kuge, Yujiro Asada

**Affiliations:** 1 Department of Pathology, University of Miyazaki, Miyazaki, Japan; 2 Department of Tracer Kinetics & Bioanalysis, Hokkaido University, Sapporo, Japan; 3 Department of Internal Medicine, Faculty of Medicine, University of Miyazaki, Miyazaki, Japan; 4 Frontier Science Research Center, University of Miyazaki, Miyazaki, Japan; 5 Faculty of Health Sciences, Institute of Medical, Pharmaceutical and Health Sciences, Kanazawa University, Ishikawa, Japan; 6 Biomedical Imaging Research Center, University of Fukui, Fukui, Japan; 7 Department of Nuclear Medicine, Graduate School of Medicine, Hokkaido University, Sapporo, Japan; 8 Central Institute of Isotope Science, Hokkaido University, Sapporo, Japan; National Cerebral and Cardiovascular Center, Japan

## Abstract

**Aims:**

Inflammation and possibly hypoxia largely affect glucose utilization in atherosclerotic arteries, which could alter many metabolic systems. However, metabolic changes in atherosclerotic plaques remain unknown. The present study aims to identify changes in metabolic systems relative to glucose uptake and hypoxia in rabbit atherosclerotic arteries and cultured macrophages.

**Methods:**

Macrophage-rich or smooth muscle cell (SMC)-rich neointima was created by balloon injury in the iliac-femoral arteries of rabbits fed with a 0.5% cholesterol diet or a conventional diet. THP-1 macrophages stimulated with lipopolysaccharides (LPS) and interferon-γ (INFγ) were cultured under normoxic and hypoxic conditions. We evaluated comprehensive arterial and macrophage metabolism by performing metabolomic analyses using capillary electrophoresis-time of flight mass spectrometry. We evaluated glucose uptake and its relationship to vascular hypoxia using ^18^F-fluorodeoxyglucose (^18^F-FDG) and pimonidazole, a marker of hypoxia.

**Results:**

The levels of many metabolites increased in the iliac-femoral arteries with macrophage-rich neointima, compared with those that were not injured and those with SMC-rich neointima (glycolysis, 4 of 9; pentose phosphate pathway, 4 of 6; tricarboxylic acid cycle, 4 of 6; nucleotides, 10 of 20). The uptake of ^18^F-FDG in arterial walls measured by autoradiography positively correlated with macrophage- and pimonidazole-immunopositive areas (r = 0.76, and r = 0.59 respectively; n = 69 for both; p<0.0001). Pimonidazole immunoreactivity was closely localized with the nuclear translocation of hypoxia inducible factor-1α and hexokinase II expression in macrophage-rich neointima. The levels of glycolytic (8 of 8) and pentose phosphate pathway (4 of 6) metabolites increased in LPS and INFγ stimulated macrophages under hypoxic but not normoxic condition. Plasminogen activator inhibitor-1 protein levels in the supernatant were closely associated with metabolic pathways in the macrophages.

**Conclusion:**

Infiltrative macrophages in atherosclerotic arteries might affect metabolic systems, and hypoxia but not classical activation might augment glycolytic and pentose phosphate pathways in macrophages.

## Introduction

Inflammation is considered a feature of atherosclerosis, and inflammation in atherosclerotic plaques is associated with plaque instability and future thrombotic complications [1]. Positron emission tomography (PET) imaging using [^18^F]-fluorodeoxyglucose (^18^F-FDG) is as a non-invasive diagnostic modality that can directly measure arterial inflammation in patients at high risk for atherosclerosis [2]. The cellular accumulation of ^18^F-FDG reflects metabolic activity because ^18^F-FDG is a direct surrogate of glucose uptake. In addition, glucose utilization and O_2_ uptake/mg DNA are increased in rabbit and monkey atherosclerotic aortae [3]. On the other hand, adenosine triphosphate (ATP) depletion in the cores of advanced rabbit atherosclerotic plaques is associated with hypoxic areas [4]. Because hypoxia can affect cellular metabolism and inflammation [5], metabolism might differ between atherosclerotic and normal arteries. Hypoxia is a potent and sufficient stimulus for increased glucose uptake in macrophages [6]. However, little comprehensive knowledge is available about the metabolic status of atherosclerotic arteries.

Metabolome analysis has been widely applied to understand biological traits and identify novel biomarkers and several clinical studies of metabolomes have revealed plasma metabolites that are related to cardiovascular risk factors, myocardial injury and the development of diabetes. [7]–[9]. Capillary electrophoresis/mass spectrometry (CE-MS) can separate metabolites with high-resolution and quantify virtually all charged low-molecular weight compounds [10]. We analyzed the metabolomes of rabbit atherosclerotic arteries using capillary electrophoresis-time of flight mass spectrometry (CE-TOFMS) to identify comprehensive metabolic changes in atherosclerosis. We also assessed the relationship between ^18^F-FDG uptake and vascular hypoxia in rabbits and cultured macrophages.

## Materials and Methods

### Ethical Statement

The Animal Care Committee of Miyazaki University and Hokkaido University approved the animal research protocols (permit number 2010-511), which also conformed to the Guide for the Care and Use of Laboratory Animals published by the US National Institutes of Health. All efforts were made to minimize suffering.

### Experimental protocol

Twenty-two male Japanese white rabbits weighing 2.5 to 3.0 kg were fed with a conventional or 0.5% cholesterol diet and then atherosclerotic lesions were induced in the right iliac-femoral arteries of all of them using a balloon catheter. Three weeks later, the rabbits were assigned to three groups for histological and metabolome (n = 6 each) analyses and an ^18^F-FDG uptake study (n = 10).

### Atherosclerosis model

Surgery proceeded under aseptic conditions and general anesthesia was achieved via an intravenous injection of pentobarbital (25 mg/kg). An angioplasty balloon catheter (diameter, 2.5 mm; length, 9 mm; QUANTUM, Boston Scientific, Galway, Ireland) was inserted via the carotid artery into the right femoral artery under fluoroscopic guidance one week after feeding with a conventional or a 0.5% cholesterol diet to induce atherosclerotic lesions in the right iliac-femoral artery. The catheter was inflated to 1.5 atm and retracted three times to denude the endothelium [11]. Three weeks later, the rabbits were fasted for six hours, and injected with heparin (500 U/kg, i.v.) and sacrificed with an overdose of pentobarbital (60 mg/kg, i.v.). The animals were perfused with 50 mL of saline for the metabolome analysis, and with 0.01 mol/L of phosphate buffered saline and 50 mL of 4% paraformaldehyde for the histological analysis. The animals involved in the ^18^F-FDG uptake study were not perfused, because a preliminary study showed that perfusion reduces arterial radioactivity levels.

### Cell culture

Human THP-1 cells (Dainippon Sumitomo Pharm., Suita, Japan) were cultured in RPMI1640 (Dainippon Sumitomo Pharm.) supplemented with 10% heat-inactivated fetal bovine serum, penicillin (100 U/ml), and streptomycin (100 µg/ml). THP-1 cells (1.0×10^5^ cells/cm^2^) were differentiated into macrophages using phorbol 12-myristate 13-acetate (PMA, 320 nM, Sigma, Saint Louis, MO, USA), and polarized as previously reported [12]. For M1 polarization, cells were treated with PMA for 6 hours and then cultured with PMA plus lipopolysaccharide (LPS, O111: B4, 10 ng/ml, Sigma-Aldrich, Saint Louis) and interferon-γ (INFγ, 20 ng/ml, R & D Systems, Minneapolis, MN, USA) for another 42 hours. For control macrophages, THP-1 cells were cultured with PMA for 48 hours. To evaluate the appropriate induction of polarization, we measured expression of M1 marker genes, interleukin (IL)-6, tumor necrosis factor (TNF)-α, and IL-1β. After replacement of culture medium, PMA-treated control macrophages or M1 polarized macrophages were incubated under normoxic (21% O_2_) or hypoxic (1% O_2_) conditions for 6 hours for metabolome analysis. We measured tissue factor (TF) and plasminogen activator inhibitor-1 (PAI-1) levels in the supernatant with Elisa kits (Quantikine ELISA for human TF and PAI-1, R & D Systems, Inc., Minneapolis, MN, USA), and analyzed correlation between these levels and metabolite levels in macrophages.

### Metabolomic analysis

Regarding rabbit arterial tissue, surrounding soft tissue was removed and then the arteries were dissected out, frozen in liquid nitrogen and stored at −80°C. Metabolites were extracted from arterial samples (42–70 mg) as follows. Samples were immersed in 500 µL of methanol containing 50 µM Internal Standard Solution 1 (Solution ID: H3304-1002, Human Metabolome Technologies, Tsuruoka, Japan) at 0°C and then homogenized three times at 1,500 rpm for 120 seconds to inactivate enzymes. The homogenate was then mixed with 200 µL of Milli-Q water and 500 µL of chloroform and centrifuged at 2,300×*g* for 4 minutes at 4°C. The upper aqueous layer (400 µL) was centrifugally filtered through a 5-kDa cutoff filter (Millipore) to remove proteins and then the filtrate was lyophilized and suspended in 50 µL of Milli-Q water.

Regarding the cell culture experiment, adherent cells on the dishes were washed with 5% mannitol aqueous solution at room temperature. The cells were immersed in 400 µL of methanol for 30 seconds, and 275 µL of the Internal Standard Solution (10 µM, Solution ID: H3304-1002, Human Metabolome Technologies) for 30 seconds. The extraction liquid was centrifuged at 2,300×*g* for 5 minutes at 4°C. The supernatant (400 µL) was centrifugally filtered at 9,100×*g* for 4 hours at 4°C through a 5-kDa cutoff filter (Millipore) to remove proteins, and then the filtrate was lyophilized and suspended in 25 µL of Milli-Q water.

The metabolite suspension was analyzed by CE-TOFMS using an Agilent capillary electrophoresis (CE) system equipped with an Agilent 6210 TOFMS, an 1100 isocratic high-performance liquid chromatography pump, a G1603A CE-MS adapter kit and a G1607A CE-electrospray ionization-mass spectrometry (ESI-MS) sprayer kit (Agilent Technologies, Waldbronn, Germany). The system was controlled using G2201AA ChemStation software version B.03.01 for CE (Agilent).

Cationic metabolites were analyzed using a fused silica capillary (50 µm i.d.×80 cm total length), with commercial cation electrophoresis buffer (Solution ID: H3301-1001, Human Metabolome Technologies) as the electrolyte. The sample was injected at a pressure of 50 mbar for 10 seconds (approximately 10 nL). The applied voltage was set at 27 kV. ESI-MS proceeded in the positive ion mode and the capillary was set at 4,000 V. The spectrometer scanned samples from 50 to 1,000 *m/z*. Other conditions were as described [13].

Anionic metabolites were analyzed using a fused silica capillary (50 µm i.d.×80 cm length), with a commercial anionic electrophoresis buffer (Solution ID: H3302-1021, Human Metabolome Technologies) as the electrolyte. The sample was injected at a pressure of 50 mbar for 25 sec (approximately 25 nL). The applied voltage was 30 kV. ESI-MS was conducted in the negative ion mode, and the capillary was set at 3,500 V. The spectrometer scanned samples from 50 to 1,000 *m/z*. Other conditions were as described [14].

Raw data obtained by CE-TOFMS were processed using MasterHands [15]. Signal peaks corresponding to isotopomers, adduct ions and other product ions of known metabolites were excluded, all signal peaks potentially corresponding to authentic compounds were extracted, and then their migration times (MT) were normalized against those of the internal standards. Thereafter, peaks were aligned according to the *m/z* and normalized MT values. Finally, peak areas were normalized against those of the internal standards, methionine sulfone and D-Camphor-10-sulfonic acid, for cations and anions, respectively. The resultant relative area values were further normalized by sample weight. Annotation tables were produced from CE-ESI-TOFMS values for standard compounds, and aligned with datasets according to similar *m/z* and normalized MT values.

### ^18^F-FDG uptake and hypoxia

We investigated ^18^F-FDG uptake as described with minor modifications [16]. Rabbits (n = 10) were fasted for four hours before being infused with ^18^F-FDG (average, 207 MBq/rabbit) and 60 mg/kg of the hypoxia marker, pimonidazole (Hypoxiaprobe-1, Natural Pharmacia International Inc., Burlington, MA, USA). Two hours later, amounts of radioactivity in the iliac-femoral arteries, and blood were measured using a well-type γ-counter (1480 WIZARD 3; Wallac Co. Ltd., Turku, Finland). The results are calculated as (%ID/g)×kg.

The non-injured or injured iliac-femoral arteries were excised, and cut into 9–10 segments or 13–15 segments in both diet groups, respectively. The arterial segments were embedded in Tissue-Tek (Sakura, Tokyo, Japan) and frozen. Consecutive 10- or 5-μm slices were prepared for autoradiography or histological analysis, respectively, and then 10-μm cryostat sections were exposed to phosphor imaging plates (Fuji Imaging Plate BAS-SR 2025, Fuji Photo Film Co. Ltd., Tokyo, Japan) for 12 h together with a set of calibrated standards [17]. The plates were then scanned using a Fuji Bio-imaging Analyzer BAS-5000 with an internal resolution of 25-μm (Fuji Photo Film) and the images were assessed using Multi Gauge Ver. 3.0 image analysis software (Fuji Photo Film). The amount of radioactivity in each image is expressed as photostimulated luminescence per unit area (PSL = a×D×t, where a is a constant, D is the amount of radioactivity exposed on the image plate, and t is exposure time). Each PSL value/mm^2^ from the arterial tissue was recorded and converted to a ratio (%) of the activity of the standard injected dose/mm^2^ of lesion area (% ID/m^2^). The data were normalized according to the weight of each rabbit (%ID/m^2^)×kg.

We measured FDG uptake in areas with or without macrophages-rich area or hypoxia to determine whether or not hypoxia augments FDG uptake in the rabbit iliac-femoral artery with macrophage-rich neointima. Areas that were rich in macrophages or pimonidazole were traced on immunohistochemical images, and then FDG uptake was measured in corresponding areas of autoradiographic images.

### Histological analysis

Excised iliac-femoral arteries were fixed in 4% paraformaldehyde for 12 hours at 4°C and embedded in paraffin. Sections 3 µm thick were stained with hematoxylin and eosin and immunohistochemically assessed using antibodies against muscle actin (HHF35; DAKO, Glostrup, Denmark), rabbit macrophages (RAM11; DAKO), CD163 (AM-3K, Trans Genic Inc. Kobe, Japan), Ki-67 (MIB-1; DAKO) and hypoxia inducible factor (HIF)-1α (H1alpha67; Abcam, Cambridge, MA, USA).

Consecutive 5-μm slices used in the ^18^F-FDG uptake study were stained with hematoxylin and eosin. Immunohistochemical assessment using antibodies against muscle actin (HHF35; DAKO), rabbit macrophages (RAM11; DAKO), pimonidazole (Natural Pharmacia International Inc.), or hexokinase-II (Abcam) was followed by staining with Envision (DAKO). Horseradish peroxidase activity was visualized using 3, 3′-diaminobenzidine tetrahydrochloride. Immunostaining controls included non-immune mouse IgG instead of primary antibodies. Areas of positive immunostaining in vessels were analyzed using the WinRoof color imaging morphometry system (Mitani, Fukui, Japan) [18]. Data are expressed as immunopositive areas (μm^2^), ratios of vascular areas (%) or numbers of immunopositive nuclei/mm^2^.

For double immunofluorescence, antibodies for rabbit macrophage (RbM2, TransGenic Inc. Kobe, Japan) and smooth muscle actin (1A4, Novus biologicals, Littleton, CA, USA) were labeled with Mix-n-Stain CF594 antibody labeling kit (Biotium, Hayward, CA, USA). The frozen sections of arteries at 3 weeks after balloon injury in rabbits fed with 0.5% cholesterol diet were stained with CF488A labeled secondary antibody (Biotium) for Ki-67 and CF594 labeled antibodies for rabbit macrophage or smooth muscle actin.

### Quantitative real-time polymerase chain reaction

Cellular total RNA isolated using Trizol (Life Technologies, Carlsbad, CA, USA) and PureLink™ RNA mini kit (Life Technologies), and converted into complementary DNA (cDNA) using the Primescript RT Mater Mix kit (Takara Bio, Otsu, Japan). cDNA were quantified by quantitative polymerase chain reaction (qPCR) on an LightCycler 480 apparatus (Roche Applied Science, Penxberg, Germany) using SYBR Premix Ex Tag™ kit (Takara Bio) and specific primers indicated in table S1. Messenger RNA (mRNA) levels were subsequently normalized to those of β-actin.

### Statistical analysis

All data are presented as means and standard deviation. Differences between individual groups were tested using the one way analysis of variance with the Bonferroni multiple comparison test (GraphPad Prizm 4.03; GraphPad Software Inc., San Diego, CA, USA). Biologically meaningful patterns were identified by metabolite set enrichment analysis using MetaboAnalyst 2.0 [19]. Relationships between factors were evaluated using Pearson's test and *P*<0.05 was considered statistically significant.

## Results

### SMC-rich or macrophage-rich neointimal lesions in rabbit iliac-femoral arteries

**Figure 1:**
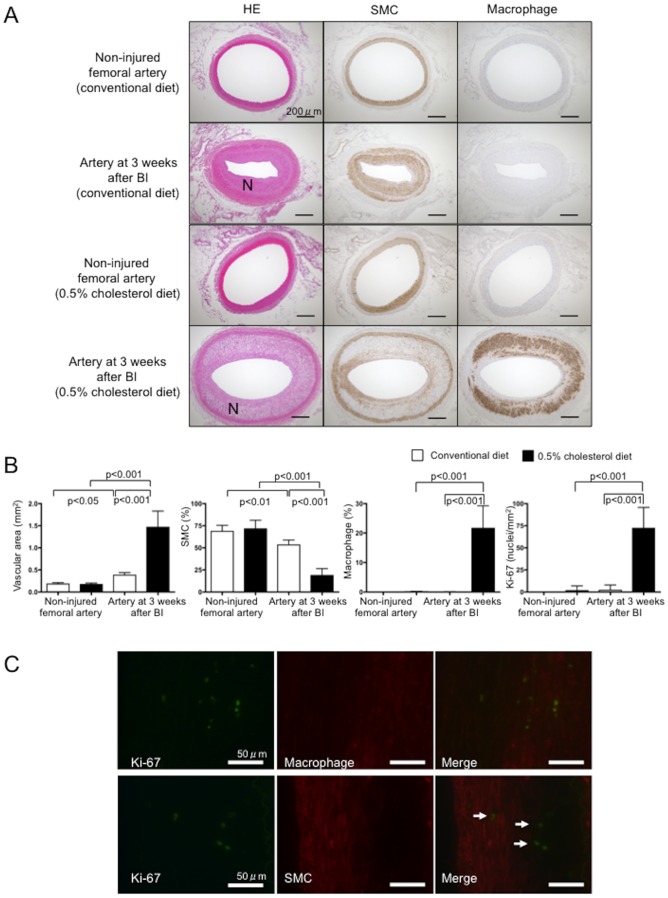
Histological findings of iliac-femoral arteries that were not injured or those at three weeks after balloon injury in rabbits fed with conventional or 0.5% cholesterol diets. A. Representative histological images of arterial sections stained with hematoxylin eosin (HE), and antibodies for muscle actin or rabbit macrophages. N, neointima; BI, balloon injury. B. Vascular areas, as well as areas that are immunopositive for SMCs and macrophages, and numbers of Ki-67-immunopositive nuclei in arteries. White and black bars, conventional and 0.5% cholesterol diet, respectively; n = 12 each). BI, balloon injury. Arteries at three weeks after balloon injury shows neointimal formation rich in SMCs (conventional diet) or macrophages (0.5% cholesterol diet), compared with non-injured arteries. C. Representative double immunofluorescent images for Ki-67 and macrophage or SMC in arteries 3 weeks after balloon injury in rabbits fed with 0.5% cholesterol diet. Images stained with CF488A labeled anti-Ki-67 antibody (green), CF495-labeled anti-macrophage antibody (red) or anti-SMC antibody (red), and merged immunofluorescent images. There are Ki-67 immunopositive nuclei in macrophages (upper row). The cells with Ki-67-immunopositive nuclei don't have SMC-positive cytoplasm (arrows).

Figure 1 shows representative histological images acquired three weeks after injuring the iliac-femoral arteries of rabbits fed with a conventional or 0.5% cholesterol diet. Balloon injury induced the formation of neointima comprising SMCs, a few macrophages and extracellular matrix in rabbits that were fed with the conventional diet (Fig. 1A), and larger neointima comprising SMCs, more macrophages and more extracellular matrix in those fed with the 0.5% cholesterol diet (Fig. 1A). The ratios of macrophages and SMC areas in the injured arteries were significantly larger and smaller, respectively in rabbits fed with the 0.5% cholesterol diet compared with the conventional diet (Fig. 1B). Thus, we defined injured iliac-femoral arteries in rabbits fed with a conventional diet or a 0.5% cholesterol diet as having SMC-rich or macrophage-rich neointima, respectively. CD163 positive area accounted for 0.34±0.22% (n = 12) in the arteries with macrophage-rich neointima. Ratio of CD163 positive area to RAM11 positive area was 0.02±0.01 (n = 12) in the arteries with macrophage-rich neointima. The number of Ki-67 immunopositive nuclei was increased in the arteries with macrophage-rich neointima (Fig. 1B), indicating increased proliferative activity. Double immunofluorescence showed Ki-67 immunopositive nuclei in macrophages but not in SMCs (Fig. 1C). Macrophage accounted for 80.4±14.4% of Ki-67 positive cells. The result suggests that proliferative cells are predominantly macrophages in the arteries.

### Metabolome analysis in rabbit iliac-femoral arteries

**Figure 2:**
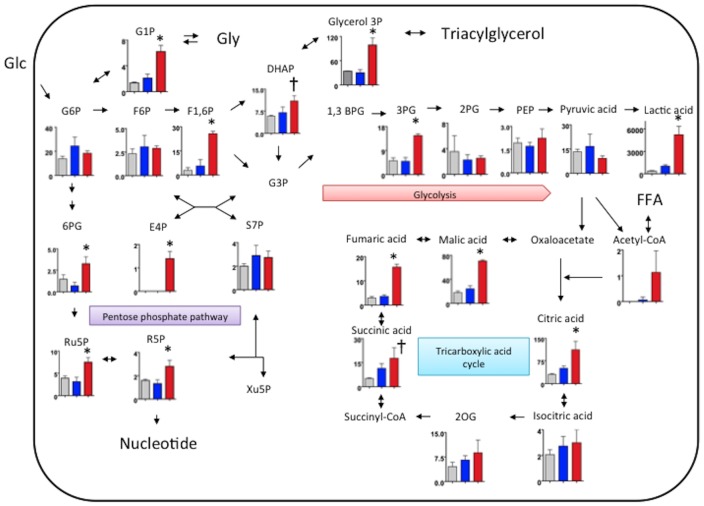
Levels of metabolites of glycolysis, the pentose phosphate pathway, tricarboxylic acid cycle and glyconeogenesis/glycogenolysis in rabbit iliac-femoral arteries. Gray, blue and red bars: iliac-femoral arteries that were not injured (conventional diet), and those with SMC-rich (conventional diet) and macrophage-rich (0.5% cholesterol diet) neointima, respectively (n = 3 for all). Metabolite levels are expressed as nmol/g. *p<0.05 vs. other groups, ^†^p<0.05 vs. non-injured femoral artery. 1,3BPG, 1,3-bisphosphoglycerate; 2PG, 2-phosphoglyceric acid; 3PG, 3-phosphoglyceric acid; 2OG, 2-oxoglutaric acid; 6PG, 6-phosphogluconic acid; DHAP, dihydroxyacetone phosphate; E4P, erythrose 4-phosphate; F1-6P, fructose 1,6-diphosphate; F6P, fructose 6-phosphate; FFA, free fatty acid; G1P, glucose 1-phosphate; G3P, glyceraldehyde 3-phosphate; G6P, glucose 6-phosphate; Glu, glucose; Gly, glycogen; PEP, phosphoenolpyruvic acid; R5P, ribose 5-phosphate; Ru5P, ribulose 5-phosphate; S7P, sedoheptulose 7-phosphate; Xu5P, xylulose 5-phosphate.

We comprehensively evaluated metabolism in non-injured iliac-femoral arteries of rabbits fed with a conventional diet, and in iliac-femoral arteries with SMC- and macrophage-rich neointima by metabolomic analysis using CE-TOFMS. Figure 2 shows levels of central carbon metabolites in the arteries. Levels of 4 of 9 glycolytic, 4 of 6 pentose phosphate cycle, and 4 of 6 citric acid cycle metabolites were increased in the arteries with macrophage-rich neointima compared with the arteries that were not injured and those with SMC-rich neointima. Levels of glucose 1-phosphate, an intermediate of glyconeogenesis/glycogenolysis, and glycerol 3-phosphate, an intermediate of triacylglycerol/glycerophospholipids, also increased in the arteries with macrophage-rich neointima. The metabolite levels shown in Figure 2 did not significantly differ between the arteries that were not injured and those with SMC-rich neointima from rabbits fed with a conventional diet.

Table S2 shows the levels of all metabolites analyzed in this study. In addition to the above, levels of 10 of 20 nucleotides, 2-oxoisovaleric acid, 2-hydroxybutyric acid, 3-hydroxybutyric acid, and glycolic acid were increased in the arteries with macrophage-rich neointima, compared with those that were not injured and those with SMC-rich neointima. Levels of nicotinamide adenine dinucleotide and nicotinamide adenine dinucleotide phosphate were also increased in the arteries with macrophage-rich neointima.

Levels of gluconic acid were significantly decreased in the arteries with SMC- and macrophage-rich neointima, compared with those that were not injured (Table S2).

Analysis of metabolite set enrichment revealed significant changes in glycolysis (p<0.001), the pentose phosphate pathway (p<0.0001), the tricarboxylic acid (TCA) cycle (p<0.001), purine metabolism (p<0.001), ribonucleic acid (RNA) transcription (p<0.00001) and pyrimidine metabolism (p<0.05) in the arteries with macrophage-rich neointima, compared with those that were not injured (conventional diet) and those with SMC-rich neointima.

### ^18^F-FDG uptake and its relationship to hypoxia in rabbit iliac-femoral arteries

**Figure 3:**
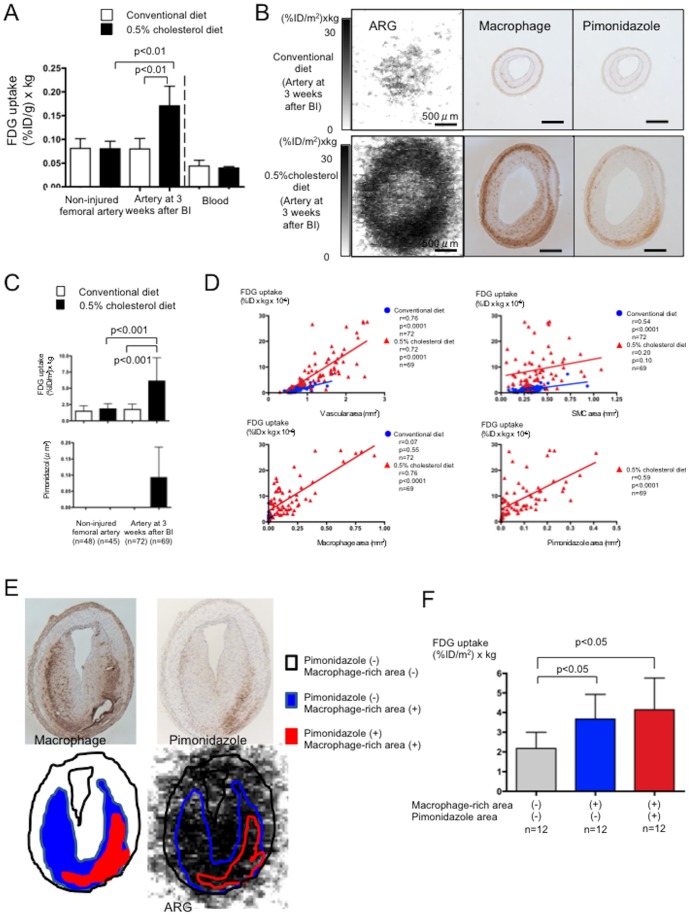
Arterial ^18^F-FDG uptake and its relationship to hypoxia. A. Radioactivity levels in iliac-femoral arteries and blood. White and black bars, conventional and 0.5% cholesterol diets, respectively; n = 5). BI, balloon injury. B. Representative findings of macrophages and pimonidazole in the iliac-femoral arteries at three weeks after balloon injury in rabbits fed with conventional (upper row) or 0.5% cholesterol diet (lower row). Autoradiographic images show increased ^18^F-FDG uptake in the artery with macrophage-rich neointima. Immunohistochemical staining shows hypoxic area (pimonidazole positive area) localized in macrophage-rich area in deep portion of wall. C. Uptake of ^18^F-FDG and immunopositive area for pimonidazole in arterial sections. White and black bars, conventional and 0.5% cholesterol diets, respectively. D. Correlations between ^18^F-FDG uptake and vascular area, SMC-, macrophage-, and pimonidazole-immunopositive areas in sections of arteries at three weeks after BI. E. Representative trace of macrophage-rich and/or pimonidazole immunopositive area in autoradiographic image. Areas that were rich in macrophages or pimonidazole were traced on immunohistochemical images, and then FDG uptake was measured in corresponding areas of autoradiographic images. F. Autoradiogram of arteries with macrophage-rich neointima shows ^18^F-FDG uptake in areas with or without macrophage-rich area or pimonidazole immunopositivity.

We evaluated glucose uptake and its relationship to vascular hypoxia by injecting rabbits with both ^18^F-FDG and the hypoxia marker, pimonidazole. Figure 3A shows the amount of radioactivity in excised arteries two hours after ^18^F-FDG infusion. Significantly more radioactivity was found in the iliac-femoral arteries with macrophage-rich neointima (0.171±0.041 (%ID/g)×kg, p<0.001) than in the non-injured iliac-femoral arteries of rabbits fed with either the conventional (0.081±0.02 (%ID/g)×kg) or the 0.5% cholesterol (0.080±0.016% (%ID/g)×kg) diet, or iliac-femoral arteries with SMC-rich neointima (0.080±0.022 (%ID/g)×kg; n = 5 each). Less radioactivity was found in the blood than in the arteries, and did not differ between conventional and 0.5% cholesterol diets (0.044±0.012 vs. 0.040±0.003 (%ID/g)×kg; Fig. 3A). Serial sections were immunohistochemically stained with antibodies for pimonidazole, the SMC marker HHF35 and the macrophage marker RAM11. Figure 3B shows autoradiographic and corresponding histological images of the arteries with SMC- and macrophage-rich neointima. Significantly more tissue radioactivity was found in the sections of arteries with macrophage- than SMC-rich neointima (Fig. 3B and C). Areas containing cells that were immunopositive for pimonidazole were localized deep in the wall and such cells were distributed in macrophage-rich areas. However, macrophages were not always immunopositive for pimonidazole. No cells were immunopositive for pimonidazole in the non-injured arteries of rabbits fed with both diets and the arteries of rabbits with SMC-rich neointima (Fig. 3B and C).

Figure 3D shows relationships between ^18^F-FDG uptake and vascular, SMC, macrophage and hypoxic areas in the arteries with SMC- or macrophage-rich neointima. Vascular areas significantly correlated with ^18^F-FDG uptake in the arteries with macrophage-rich (r = 0.72, p<0.0001, n = 69) and SMC-rich (r = 0.76, p<0.0001, n = 76) neointima. However, more ^18^F-FDG was uptaken by the arteries with macrophage- than SMC-rich neointima. The SMC areas correlated with ^18^F-FDG uptake in the arteries with SMC (r = 0.54, p<0.0001, n = 72)- but not macrophage (r = 0.20, p = 0.10, n = 69) -rich neointima. The uptake of ^18^F-FDG significantly correlated with macrophage (r = 0.76, p<0.0001, n = 69) and hypoxic (r = 0.59, p<0.0001, n = 69) areas in the arteries with macrophage-rich neointima. A few macrophages were evident in those with SMC-rich neointima, but areas of macrophages did not correlate with FDG uptake (r = 0.07, p = 0.55, n = 72).

We measured ^18^F-FDG uptake in areas with or without macrophage-rich area or hypoxia to determine whether hypoxia augments ^18^F-FDG uptake in rabbit iliac-femoral arteries with macrophage-rich neointima (Fig. 3E). More ^18^F-FDG was uptaken in areas with, than without macrophages-rich area (3.8±1.3 vs. 2.2±0.8 (%ID/m^2^)×kg; n = 12 each; p<0.001). ^18^F-FDG uptake in macrophage-rich area tended more in hypoxic area, but did not significantly differ from non-hypoxic area (4.1±1.6 vs. 3.7±1.3 (%ID/m^2^)×kg; n = 12 each; Fig. 3F).

### Nuclear localization of HIF-1α and expression of HK-II in iliac-femoral arteries

**Figure 4:**
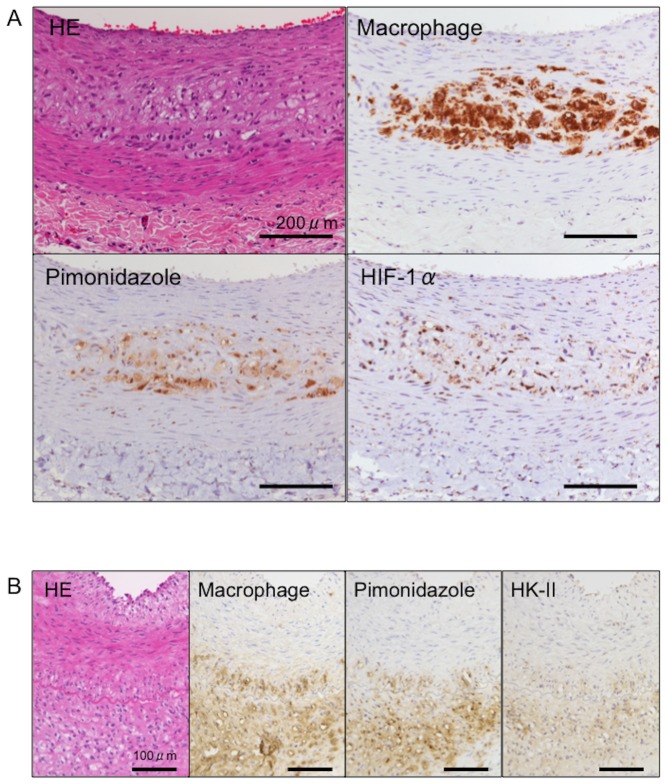
Immunohistochemical findings of HIF-1α and HK-II in iliac-femoral artery with macrophage-rich neointima. Immunohistochemical staining for macrophages, pimonidazole and HIF-1α in paraffin sections (A) and for macrophages, pimonidazole and HK-II in frozen sections (B)

We examined the nuclear localization of HIF-1α and the expression of HK-II, a glycolysis enzyme induced by HIF-1 to evaluate hypoxic responses in iliac-femoral arteries. Due to the transitory nuclear localization of HIF-1α [20], we immunohistochemically stained HIF-1α in 4% paraformaldehyde fixed-paraffin embedded sections. Figure 4 shows immunohistochemical images of RAM11, pimonidazole, HIF-1α, and HK-II staining in the arteries with macrophage-rich neointima. Areas that were positive for nuclei containing HIF-1α and HK-II closely localized with those that were positive for macrophages and pimonidazole. No nuclei immunopositive for HIF-1α or HK-II immunoreactivity were evident in the non-injured arteries from rabbits fed with conventional or 0.5% cholesterol diets or in the arteries with SMC-rich neointima.

### Metabolome analysis in cultured macrophages

Macrophages treated with LPS and INFγ showed significant upregulation of IL-6 (control: 0.12±0.03, M1: 0.84±0.52, p<0.01, n = 6), TNF-α (control: 0.34±0.04, M1: 0.90±0.15, p<0.0001, n = 6), and IL-1β mRNA (control: 4.43±0.57, M1: 6.24±0.18, p<0.0001, n = 6) compared with PMA-treated control macrophages.

**Figure 5:**
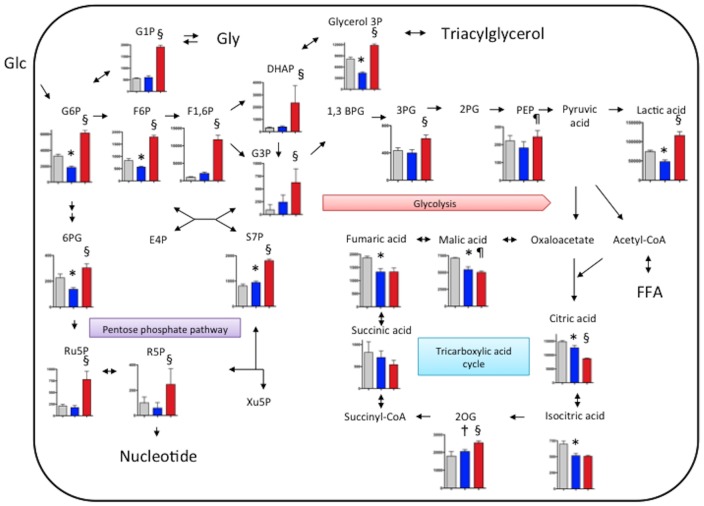
Levels of metabolites of glycolysis, the pentose phosphate pathway, tricarboxylic acid cycle and glyconeogenesis/glycogenolysis in cultured macrophages. For M1 polarization, THP-1 cells were treated with PMA (320 nM) for 6 hours and then cultured with PMA plus LPS (10 ng/ml) and INFγ (20 ng/ml,) for another 42 hours. After replacement of culture medium, PMA-treated control macrophages (gray bar, n = 6) and M1 macrophages (blue bar, n = 6) were incubated under normoxic (21% O_2_) condition for 6 hours, or the M1 macrophages were incubated hypoxic (1% O_2_) conditions for 6 hours (red bar, n = 6). Metabolite levels are expressed as pmol/10^6^ cell. *p<0.01 vs. PMA control, ^†^p<0.05 vs. PMA control, ^§^p<0.01 vs. M1 normoxia, ^¶^p<0.05 vs. M1 normoxia. 1,3BPG, 1,3-bisphosphoglycerate; 2PG, 2-phosphoglyceric acid; 3PG, 3-phosphoglyceric acid; 2OG, 2-oxoglutaric acid; 6PG, 6-phosphogluconic acid; DHAP, dihydroxyacetone phosphate; E4P, erythrose 4-phosphate; F1-6P, fructose 1,6-diphosphate; F6P, fructose 6-phosphate; FFA, free fatty acid; G1P, glucose 1-phosphate; G3P, glyceraldehyde 3-phosphate; G6P, glucose 6-phosphate; Glu, glucose; Gly, glycogen; PEP, phosphoenolpyruvic acid; R5P, ribose 5-phosphate; Ru5P, ribulose 5-phosphate; S7P, sedoheptulose 7-phosphate; Xu5P, xylulose 5-phosphate.

To evaluate comprehensive metabolic status in macrophages, we performed metabolomic analysis with CE-TOFMS in PMA-treated control and M1 polarized macrophages under normoxic condition or M1 polirized macrophages under hypoxic (1% O_2_) conditions for 6 hours. Table S3 shows levels of 110 metabolites in the cultured macrophages. Under normoxic condition, 42 metabolite levels significantly differed between control and M1 polirized macrophages. Under hypoxic condition, 54 metabolite levels significantly differed in M1 polarized macrophages. Figure 5 shows levels of central carbon metabolites in the macrophages. Levels of 3 of 8 glycolytic, 2 of 4 pentose phosphate cycle metabolites, and 5 of 6 TCA cycle metabolites were altered in M1 polarized macrophages compared with those in control macrophages. The metabolite levels of TCA cycle except for 2-oxoglutalic acid decreased in M1 polarized macrophages. In M1 polarized macrophages, the hypoxic condition increased in levels of all glycolytic-, all pentose phosphate cycle metabolites, glucose 1-phosphate, and glycerol 3-phosphate (Fig.5). In addition, the hypoxic condition affected one-half of amino acid levels and one-third of nucleotide levels (Table S3).

### Relation between TF or PAI-1 and metabolite levels in cultured macrophages

We assessed the correlation between TF or PAI-1 levels in the supernatant and metabolite levels in the macrophages. The TF levels negatively correlated with macrophage creatine level (r = −0.495, p<0.05, n = 18). While, the PAI-1 levels correlated with one-half of the metabolite levels in macrophages. Moreover, the correlation tended a similar manner within the pathways or metabolite groups. The PAI-1 levels positively correlated with TCA cycle metabolites and nucleotide, and negatively correlated with glycolysis, pentose phosphate pathway metabolites, and amino acids (Table S4).

## Discussion

The CE-TOFMS and ^18^F-FDG findings showed increased levels of metabolites in the glycolytic and pentose phosphate pathways and the TCA cycle, as well as of nucleotides in macrophage-rich atherosclerotic iliac-femoral arteries from rabbits. We also found that hypoxia affects the levels of glycolytic and pentose phosphate pathways in M1 polarized macrophages, and that PAI-1 protein levels are closely associated with metabolic pathways in macrophages.

Clinical and preclinical studies have positively associated increased ^18^F-FDG uptake in atherosclerotic arteries [21] with macrophage density [22]. Morrison et al. [3] examined glucose utilization in rabbit and rhesus monkey aortic atherosclerosis induced by high-fat diets, and found increased glucose utilization and degradation to lactic acid in atherosclerotic intima and media. Our results support the notion that glycolysis is enhanced in macrophage-rich atherosclerotic arteries and that it might reflect metabolic activity in macrophage-rich lesion rather than SMC-rich lesion, because levels of glycolytic metabolites, ^18^F-FDG uptake and HK-II expression in iliac-femoral arteries with SMC-rich neointima did not significantly increase (Table S2, Figs. 2 and 3). Hypoxia is a potent stimulus for increased glucose uptake in macrophages and foam cells in vitro, suggesting that hypoxia rather than pro-inflammatory cytokines augments ^18^F-FDG uptake in atherosclerotic plaque [6]. In consistent with the previous study [6], HIF-1α nuclear translocation and HK-II expression were observed in areas that were positive for pimonidazole (Fig. 4) and ^18^F-FDG uptake was positively associated with hypoxic areas (Fig. 3D). In addition, hypoxia increased in glycolytic metabolite levels in M1 polarized macrophages (Fig.5 and Table S3). However, ^18^F-FDG uptake did not significantly differ between hypoxic and non-hypoxic macrophage-rich areas of the arteries with macrophage-rich neointima (Fig. 3F). This may be due to metabolic status of SMCs, endothelial cells, and lymphocytes in macrophage-rich area. Relative reduction of blood flow in hypoxic area might affect ^18^F-FDG supply. And, the discrepancy might be due to some difference between ^18^F-FDG uptake and the levels of down stream metabolites, or special resolution of the autoradiography. Classical but not innate activation of cultured macrophages significantly increases 2-deoxy-D-glucose uptake, accompanied by glycolytic pathway activation [23]. Classical activation increases lactate production with a decrease in oxygen consumption by mitochondria. In our in vitro study, M1 polarization decreased in levels of TCA cycle metabolites but not increased in levels of glycolytic metabolites (Fig. 5). The present findings support the notion that hypoxic stimuli enhance glycolysis in macrophage-rich atherosclerotic arteries and M1 polarized macrophages. However, it remains controversial whether non-hypoxic stimuli enhance glycolysis in macrophages.

The TCA cycle is a series of chemical reactions that generates energy through the oxidization of acetate derived from carbohydrates, fats and proteins to carbon dioxide. Glucose oxidation accounts for only 10% of the total O_2_ uptake in normal rabbit and monkey aortae, whereas oxygen uptake doubles in atherosclerotic aortae, although glucose oxidation does not increase [3]. A series of studies found that almost all glucose is converted into lactate, very little is oxidized, and almost all oleate, (long-chain fatty acid) is converted into CO_2_ in peritoneal macrophages in vitro [24], [25]. More citrate synthase activity is found in macrophages than in lymphocytes [26]. Therefore, the increased level of citric acid in the arteries with macrophage-rich neointima might reflect enhanced fatty acid oxidation. On the other hand, vascular hypoxia might be associated with increased levels of malate, fumarate and succinate in femoral arteries with macrophage-rich neointima, because anaerobic mitochondrial oxidation of 2-oxoglutarate to succinate and reduction of fumarate to succinate can generate ATP and maintain mitochondrial energization [27], [28]. Taken together, increased levels of TCA intermediates could reflect enhanced aerobic fatty acid oxidation and anaerobic pathways for ATP production in macrophage-rich atherosclerotic arteries.

The pentose phosphate pathway is an alternative route of glucose catabolism that functions in the formation of ribose 5-phosphate (R5P) for synthesis of RNA and deoxyribonucleic acid, thus supporting cell growth and proliferation, as well as the formation of nicotinamide adenine dinucleotide phosphate (NADPH) for biosynthetic reactions. NADPH protects directly against oxidative stress by neutralizing reactive oxygen intermediates, and indirectly via regenerating reduced glutathione (GSH) from its oxidative form, GSSG. The amounts of glucose oxidized by the pentose phosphate pathway in arteries have been described as large and small [29], [30]. Levels of metabolites from the pentose phosphate pathway in the rabbit iliac-femoral artery were equivalent to or less than those of glycolytic metabolites. These findings suggest that the pentose phosphate pathway plays a role in atherosclerosis, and that the enhanced pentose phosphate pathway is compatible with an increase in the numbers of proliferative cells and in 2-hydroxybutyric acid levels in the arteries with macrophage-rich neointima. When cystathionine is cleaved to cysteine that is incorporated into glutathione, 2-hydroxybutyric acid is released as a by-product and it is associated with increased oxidative stress [31]. The role of the pentose phosphate pathway in atherosclerosis and its role in complicating thrombosis are poorly understood. A reduction in glucose 6-phosphate dehydrogenase activity decreases vascular superoxide and atherosclerotic lesions in mice that are deficient in apolipoprotein E [32]. The findings of one study in vitro have raised the notion that changes in glucose metabolism influence the inflammatory properties of M1 macrophages [33]. Stimulation with lipopolysaccharide (LPS), which drives an M1-like or classical activation pathway, increases glycolysis as well as metabolism via the pentose phosphate pathway and decreases oxygen consumption rates in cultured macrophages. The carbohydrate kinase-like protein that catalyzes the production of sedoheptulose-7-phosphate, an intermediate of the pentose phosphate pathway, can influence macrophage polarization and antagonize the LPS-induced production of nuclear factor-κB-regulated cytokines. In contrast to the study, hypoxia but not classical activation increased in metabolite levels of pentose phosphate pathway in cultured macrophage (Fig.5 and Table S3). Because 98% of macrophages in the femoral arteries with macrophage-rich neointima were of the non-M2 type, increased amounts of pentose phosphate pathway metabolites might reflect their metabolic status and be affected by hypoxic milieu.

Nucleotide levels were increased in iliac-femoral arteries with macrophage-rich neointima. This finding is consistent with the increased levels of metabolites of the pentose phosphate pathway and proliferative activity in macrophage-rich atherosclerotic arteries. In contrast to our findings, one study discovered ATP depletion in atherosclerotic plaques of the rabbit aorta [4]. Obvious depletion in the cores of plaques >500 µm thick was associated with hypoxic areas and low glucose concentrations in the core. Because ^18^F-FDG uptake was increased in hypoxic areas of deep portions of plaque (Fig. 3B), a low glucose concentration in the core is less likely in this model. The difference might be due to the size of the plaque and the absence of a necrotic core in this model.

Balloon injury induces phenotypic changes in SMCs, and the neointima mainly comprised the synthetic phenotype type of SMCs [34]. Transcription, signalling pathways and protein expression significantly differ between synthetic and contractile phenotypes of SMCs [35]. Therefore, we presumed some metabolic changes between the non-injured and injured arteries of rabbits fed with conventional diet. However, the only analyzed metabolite that significantly differed was gluconic acid.

Gluconic acid is a non-toxic glucose derivative that occurs naturally in fruits and vegetables, and levels were reduced in the injured arteries of rabbits fed with either diet. Despite many commercial applications in pharmaceutical and food industries, the potential roles of gluconic acid in vivo are unclear. Saluk-Juszczak [36] investigated the effect of sodium D-gluconic acid on platelet activation and found that it inhibited thrombin-induced arachidonic peroxidation, O_2_^−i^ production and platelet oxidation/nitration induced by peroxynitrite, suggesting that it exerts antioxidant effects. Because oxidative stress plays a substantial role in atherogenesis and neointimal formation after balloon angioplasty [37], a reduction of gluconic acid in injured arteries implies an unbalanced redox state.

We found increased levels of glucose 1-phosphate, 2-oxoisovaleric acid, glycolic acid, glycerol 3-phosphate and 3-hydroxybutylic acid in arteries with macrophage-rich neointima. This indicates that other metabolic pathways, such as glyconeogenesis/glycogenolysis, and amino acids triglyceride and glycerophospholipid metabolism are altered in such an environment. A clinical PET/CT study has shown that ^11^C-acetate uptake, which reflects acetyl-CoA production, is increased in the aorta with advanced atherosclerosis, suggesting enhanced fatty acid synthesis [38]. Further comprehensive study is required to unveil other metabolic changes in atherosclerotic lesions.

Macrophages incubated in 1% oxygen showed an increased triglyceride and sterol contents though HIF-1α mediated cholesterol synthesis and reduction of ATP-binding cassette subfamily A member1 mediated cholesterol efflux [39]. Chronic hypoxia activated Akt pathway in human macrophages [40]. It is considered that Akt singling stimulates transport and metabolism of both glucose and amino acids, with in turn support mTOR-dependent increases in protein translation in cancer cells [41]. Our in vitro study showed significant metabolic change in M1 polarization and hypoxic condition in macrophages (Fig.5 and Table S3). Hypoxic and non-hypoxic stimuli may substantially modify macrophage metabolism.

In spite of the widespread of lipid lowering therapy, atherosclerotic disease remains a leading cause of death and loss of productive life. Metabolomic approach may discover a novel markers and therapeutic targets of atherosclerosis. We found that PAI-1 protein levels are closely associated with metabolic pathways in macrophages (Table S4). Furthermore, we recently demonstrated that ^18^F-FDG uptake reflect nuclear factor-κB activation, tissue factor, an initiator of coagulation cascade, expression, and arterial thrombus formation in rabbits [42]. These findings suggest a link between vascular metabolism and thrombogenic potential of atherosclerotic artery.

There are several limitations in this study. First, the metabolites in this study are a part of total vascular metabolites. Combined CE-TOFMS with liquid chromatography-mass spectrometry might unveil further comprehensive metabolic changes in atherosclerotic arteries. Second, we cannot refer to a dynamic process of vascular metabolism, because the arterial metabolite levels represent a metabolic point in rabbits fasted for 6 hours. Further studies are required to reveal a metabolic rate in the pathways and metabolic changes before and after diet in atherosclerotic artery. Third, it is possible that neointimal SMCs contribute to the metabolic changes and ^18^F-FDG uptake in arteries with macrophage-rich neointima, because inflammatory stimuli mildly enhanced glucose uptake in cultured vascular SMCs [6].

In conclusion, Infiltrative macrophages in atherosclerotic arteries might affect metabolic systems, and hypoxia but not classical activation might augment glycolytic and pentose phosphate pathways in macrophages.

## Supporting Information

Table S1 **Sequences of primers used for qPCR analysis.**
(PDF)

Table S2 **Quantities of arterial metabolites.**
(PDF)

Table S3 **Quantities of macrophage metabolites.**
(PDF)

Table S4 **Relation between plasminogen activator inhibitor-1 and metabolite levels.**
(PDF)

## References

[1] Libby P (2002) Inflammation in atherosclerosis. Nature 420: 868–874. 12490960 10.1038/nature01323

[2] Hiari N, Rudd JH (2011) FDG PET imaging and cardiovascular inflammation. Curr Cardiol Rep 13: 43–48. 20949379 10.1007/s11886-010-0150-5

[3] Morrison ES, Scott RF, Kroms M, Frick J (1972) Glucose degradation in normal and atherosclerotic aortic intima-media. Atherosclerosis 16: 175–184. 4628890 10.1016/0021-9150(72)90051-2

[4] Leppänen O, Björnheden T, Evaldsson M, Borén J, Wiklund O, et al (2006) ATP depletion in macrophages in the core of advanced rabbit atherosclerotic plaques in vivo. Atherosclerosis 188: 323–330. 16405894 10.1016/j.atherosclerosis.2005.11.017

[5] Semenza GL (2010) Oxygen homeostasis. Wiley Interdiscip Rev Syst Biol Med 2: 336–361. 20836033 10.1002/wsbm.69

[6] Folco EJ, Sheikine Y, Rocha VZ, Christen T, Shvartz E, et al (2011) Hypoxia but not inflammation augments glucose uptake in human macrophages: Implications for imaging atherosclerosis with 18fluorine-labeled 2-deoxy-D-glucose positron emission tomography. J Am Coll Cardiol 58: 603–614. 21798423 10.1016/j.jacc.2011.03.044

[7] Lewis GD, Wei R, Liu E, Yang E, Shi X, et al (2008) Metabolite profiling of blood from individuals undergoing planned myocardial infarction reveals early markers of myocardial injury. *J Clin Invest* 118: 3503–3512. 18769631 10.1172/JCI35111 PMC2525696

[8] Wang TJ, Larson MG, Vasan RS, Cheng S, Rhee EP, et al (2011) Metabolite profiles and the risk of developing diabetes. Nat Med 17: 448–453. 21423183 10.1038/nm.2307 PMC3126616

[9] Cheng S, Rhee EP, Larson MG, Lewis GD, McCabe EL, et al (2012) Metabolite profiling identifies pathways associated with metabolic risk in humans. Circulation 125: 2222–2231. 22496159 10.1161/CIRCULATIONAHA.111.067827 PMC3376658

[10] Soga T, Ohashi Y, Ueno Y, et al (2003) Quantitative metabolome analysis using capillary electrophoresis mass spectrometry. J Proteome Res 2: 488–494. 14582645 10.1021/pr034020m

[11] Yamashita A, Shoji K, Tsuruda T, Naraoka H, Tomita M, et al (2008) Medial and adventitial macrophages are associated with expansive atherosclerotic remodeling in rabbit femoral artery. Histol Histopathol 23: 127–136. 17999368 10.14670/HH-23.127

[12] Satomi T, Ogawa M, Mori I, Ishino S, Kubo K, et al (2013) Comparison of contrast agents for atherosclerosis imaging using cultured macrophages: FDG versus ultrasmall superparamagnetic iron oxide. J Nucl Med 54: 999–1004. 23670898 10.2967/jnumed.112.110551

[13] Soga T, Heiger DN (2000) Amino acid analysis by capillary electrophoresis electrospray ionization mass spectrometry. Anal Chem 72: 1236–1241. 10740865 10.1021/ac990976y

[14] Soga T, Ueno Y, Naraoka H, Ohashi Y, Tomita M, et al (2002) Simultaneous determination of anionic intermediates for Bacillus subtilis metabolic pathways by capillary electrophoresis electrospray ionization mass spectrometry. Anal Chem 74: 2233–2239. 12038746 10.1021/ac020064n

[15] Sugimoto M, Hirayama A, Ishikawa T, Robert M, Baran R, et al (2010) Differential metabolomics software for capillary electrophoresis-mass spectrometry data analysis. Metabolomics 6: 27–41.

[16] Zhao S, Kuge Y, Yi M, Zhao Y, Hatano T, et al (2011) Dynamic 11C-methionine PET analysis has an additional value for differentiating malignant tumors from granulomas: an experimental study using small animal PET. Eur J Nucl Med Mol Imaging 38: 1876–1886. 21732106 10.1007/s00259-011-1865-2

[17] Zhao Y, Zhao S, Kuge Y, Strauss WH, Blankenberg FG, et al (2011) Localization of deoxyglucose and annexin A5 in experimental atheroma correlates with macrophage infiltration but not lipid deposition in the lesion. Mol Imaging Biol 13: 712–720. 20686858 10.1007/s11307-010-0389-7

[18] Yamashita A, Matsuda S, Matsumoto T, Moriguchi-Goto S, Takahashi M, et al (2009) Thrombin generation by intimal tissue factor contributes to thrombus formation on macrophage-rich neointima but not normal intima of hyperlipidemic rabbits. Atherosclerosis 206: 418–426. 19371874 10.1016/j.atherosclerosis.2009.03.014

[19] Xia J, Mandal R, Sinelnikov IV, Broadhurst D, Wishart DS (2012) MetaboAnalyst 2.0–a comprehensive server for metabolomic data analysis. Nucleic Acids Res 40: W127–33. 22553367 10.1093/nar/gks374 PMC3394314

[20] Semenza GL (2004) O2-regulated gene expression: transcriptional control of cardiorespiratory physiology by HIF-1. J Appl Physiol 96: 1173–1177. 14766767 10.1152/japplphysiol.00770.2003

[21] Yun M, Yeh D, Araujo LI, Jang S, Newberg A, et al (2001) F-18 FDG uptake in the large arteries: a new observation. Clin Nucl Med 26: 314–319. 11290891 10.1097/00003072-200104000-00007

[22] Ogawa M, Ishino S, Mukai T, Asano D, Teramoto N, et al (2004) (18)F-FDG accumulation in atherosclerotic plaques: immunohistochemical and PET imaging study. J Nucl Med 45: 1245–1250. 15235073

[23] Tawakol A, Rudd JH, Fayad ZA, Boscá L (2012) Classical but not innate activation augments 2-deoxy-D-glucose uptake in macrophages: Implications for atherosclerosis imaging. J Nucl Med 53 S1: 141.

[24] Newsholme P, Gordon S, Newsholme EA (1987) Rates of utilization and fates of glucose, glutamine, pyruvate, fatty acids and ketone bodies by mouse macrophages. Biochem J 242: 631–636. 3593269 10.1042/bj2420631 PMC1147758

[25] Newsholme P, Newsholme EA (1989) Rates of utilization of glucose, glutamine and oleate and formation of end-products by mouse peritoneal macrophages in culture. Biochem J. 261: 211–218. 2775207 10.1042/bj2610211 PMC1138802

[26] Newsholme P, Curi R, Gordon S, Newsholme EA (1986) Metabolism of glucose, glutamine, long-chain fatty acids and ketone bodies by murine macrophages. Biochem J. 239: 121–125. 3800971 10.1042/bj2390121 PMC1147248

[27] Randall HM Jr, Cohen JJ (1966) Anaerobic CO_2_ production by dog kidney in vitro. Am J Physiol 211: 493–505. 4288380 10.1152/ajplegacy.1966.211.2.493

[28] Runeberg L, Pakarinen A (1967) Anaerobic decarboxylation of alpha-ketoglutarate by rat mitochondria with fumarate as a hydrogen acceptor. An anaerobic reaction of the respiratory chain. Ann Med Exp Biol Fenn 45: 428–433. 5602745

[29] Lofland HB Jr, Clarkson TB (1965) Certain metabolic patterns of atheromatous pigeon aortas. Arch Pathol 80: 291–296. 14322951

[30] Whereat AF (1967) Recent advances in experimental and molecular pathology. Atherosclerosis and metabolic disorder in the arterial wall. Exp Mol Pathol 7: 233–247. 4383229 10.1016/0014-4800(67)90032-9

[31] Lord RS, Bralley JA (2008) Clinical applications of urinary organic acids. Part I: Detoxification markers. Altern Med Rev 13: 205–215. 18950247

[32] Matsui R, Xu S, Maitland KA, Mastroianni R, Leopold JA, et al (2006) Glucose-6-phosphate dehydrogenase deficiency decreases vascular superoxide and atherosclerotic lesions in apolipoprotein E(-/-) mice. Arterioscler Thromb Vasc Biol 26: 910–916. 16439706 10.1161/01.ATV.0000205850.49390.3b

[33] Haschemi A, Kosma P, Gille L, Evans CR, Burant CF, et al (2012) The sedoheptulose kinase CARKL directs macrophage polarization through control of glucose metabolism. Cell Metab15: 813–826. 10.1016/j.cmet.2012.04.023 PMC3370649 22682222

[34] Kuro-o M, Nagai R, Nakahara K, Katoh H, Tsai RC, et al (1991) cDNA cloning of a myosin heavy chain isoform in embryonic smooth muscle and its expression during vascular development and in arteriosclerosis. J Biol Chem 266: 3768–3773. 1995631

[35] Kumar MS, Owens GK (2003) Combinatorial control of smooth muscle-specific gene expression. Arterioscler Thromb Vasc Biol 23: 737–747. 12740224 10.1161/01.ATV.0000065197.07635.BA

[36] Saluk-Juszczak J (2010) A comparative study of antioxidative activity of calcium-D-glucarate, sodium-D-gluconate and D-glucono-1,4-lactone in a human blood platelet model. Platelets 21: 632–640. 20873960 10.3109/09537104.2010.512210

[37] Kawamoto R, Yamashita A, Nishihira K, Furukoji E, Hatakeyama K, et al (2006) Different inflammatory response and oxidative stress in neointimal hyperplasia after balloon angioplasty and stent implantation in cholesterol-fed rabbits. Pathol Res Pract 202: 447–456. 16635553 10.1016/j.prp.2005.12.011

[38] Derlin T, Habermann CR, Lengyel Z, Busch JD, Wisotzki C, et al (2011) Feasibility of 11C-acetate PET/CT for imaging of fatty acid synthesis in the atherosclerotic vessel wall. J Nucl Med 52: 1848–1854. 22065877 10.2967/jnumed.111.095869

[39] Parathath S, Mick SL, Feig JE, Joaquin V, Grauer L, et al (2011) Hypoxia is present in murine atherosclerotic plaques and has multiple adverse effects on macrophage lipid metabolism. Circ Res 28: 1141–1152. 10.1161/CIRCRESAHA.111.246363 PMC3208906 21921268

[40] Deguchi JO, Yamazaki H, Aikawa E, Aikawa M (2009) Chronic hypoxia activates the Akt and beta-catenin pathways in human macrophages. Arterioscler Thromb Vasc Biol 29: 1664–1670. 19644051 10.1161/ATVBAHA.109.194043

[41] Plas DR, Thompson CB (2005) Akt-dependent transformation: there is more to growth than just surviving. Oncogene 24: 7435–7442. 16288290 10.1038/sj.onc.1209097

[42] Yamashita A, Zhao Y, Zhao S, Matsuura Y, Sugita C, et al (2013) Arterial 18F-Fluorodeoxyglucose Uptake Reflects Balloon Catheter-Induced Thrombus Formation and Tissue Factor Expression via Nuclear Factor-κB in Rabbit Atherosclerotic Lesions. Circ J 77: 2626–2635. 23832535 10.1253/circj.cj-12-1463

