# Supplementary material for: Increased Metabolite Levels of Glycolysis and Pentose Phosphate Pathway in Rabbit Atherosclerotic Arteries and Hypoxic Macrophage
Source: PLoS One. 2014 Jan 23;9(1):e86426. doi: 10.1371/journal.pone.0086426 (PMC3900532; doi:10.1371/journal.pone.0086426)
Supplement: Table S1 — Sequences of primers used for qPCR analysis. (PDF) [file pone.0086426.s001.pdf]

Table S1. Sequences of primers used for qPCR analysis

Gene	Forward	Reverse
Interleukine-6	5'-AAGCCAGAGCTGTGCAGATGAGTA-3'	5'-TGTCCTGCAGCCACTGGTTC-3'
Tumor necrosis factor-α	5'-TGCTTGTTCCTCAGCCTCTT-3'	5'-CAGAGGGCTGATTAGAGAGAGGT-3'
Interleukine-1β	5'-GCTGATGGCCCTAAACAGATGAA-3'	5'-TCCATGGCCACAACAACTGAC-3'
β-actin	5'-TGGCACCCAGCACAATGAA-3'	5'-CTAAGTCATAGTCCGCCTAGAAGCA-3'
