# Supplementary material for: Increased Metabolite Levels of Glycolysis and Pentose Phosphate Pathway in Rabbit Atherosclerotic Arteries and Hypoxic Macrophage
Source: PLoS One. 2014 Jan 23;9(1):e86426. doi: 10.1371/journal.pone.0086426 (PMC3900532; doi:10.1371/journal.pone.0086426)
Supplement: Table S2 — Quantities of arterial metabolites. (PDF) [file pone.0086426.s002.pdf]

Table S2 Quantities of arterial metabolites

	Metabolite	HMDB ID	Concentration (nmol/g) Conventional diet, non-injured artery (normal) n=3, mean ± SD	Conventional diet, injured artery (SMC-rich neointima) n=3, mean ± SD	0.5% cholesterol diet, injured artery (macrophage-rich neointima) n=3, mean ± SD	p value one-way ANOVA / Bonfferoni post test
Glycolysis	Glucose 6-phosphate	HMDB01401	14 ± 2	24 ± 7.4	18 ± 2.1	0.08
	Fructose 6-phosphate	HMDB00124	2.3 ± 0.5	3.0 ± 1.2	2.9 ± 0.3	0.51
	Fructose 1,6-diphosphate	HMDB01058	2.9 ± 1.7	5.6 ± 4.0	26 ± 1.5	*p<0.001
	Dihydroxyacetone phosphate	HMDB01473	5.8 ± 0.3	7.0 ± 1.9	12 ± 1.8	**p<0.05
	Glyceraldehyde 3-phosphate	HMDB01112	N.D.	N.D.	N.D.	
	3-Phosphoglyceric acid	HMDB00807	5.2 ± 1.1	5.0 ± 1.4	15 ± 0.6	*p<0.001
	2-Phosphoglyceric acid	HMDB03391	3.6 ± 2.4	2.3 ± 0.8	2.5 ± 0.4	0.56
	Phosphoenolpyruvic acid	HMDB00263	1.9 ± 0.3	1.7 ± 0.8	2.2 ± 0.6	0.35
	Pyruvic acid	HMDB00243	14 ± 1.6	16 ± 7.4	9.3 ± 1.5	0.22
	Lactic acid	HMDB00190,HMDB01311	362 ± 60	1,034 ± 169	5,235 ± 1109	*p<0.001
Pentose phosphate cycle	6-Phosphogluconic acid	HMDB01316	1.5 ± 0.6	0.7 ± 0.4	3.3 ± 0.8	*p<0.05
	Ribulose 5-phosphate	HMDB00618	3.9 ± 0.5	3.2 ± 1.0	7.5 ± 1.0	*p<0.01
	Ribose 5-phosphate	HMDB01548	1.6 ± 0.1	1.3 ± 0.4	2.8 ± 0.5	*p<0.05
	Sedoheptulose 7-phosphate	HMDB01068	2.0 ± 0.2	2.9 ± 0.8	2.7 ± 0.6	0.23
	Erythrose 4-phosphate	HMDB01321	N.D.	N.D.	1.4 ± 0.3	*p<0.001
	Phosphoribosyl pyrophosphate	HMDB00280	N.D.	N.D.	N.D.	
Tricarboxylic acid cycle	Acetyl CoA_divalent	HMDB01206	N.D.	0.07 ± 0.1	1.1 ± 0.8	0.05
	Citric acid	HMDB00094	30 ± 2.8	50 ± 8.2	112 ± 28	*p<0.05
	cis-Aconitic acid	HMDB00072	1.6 ± 0.1	2.1 ± 0.3	1.8 ± 0.4	0.25
	Isocitric acid	HMDB00193	2.1 ± 0.4	2.7 ± 0.8	3.0 ± 1.0	0.36
	2-Oxoglutaric acid	HMDB00208	4.6 ± 1.3	6.6 ± 1.3	8.8 ± 3.4	0.2
	Succinic acid	HMDB00254	5.2 ± 0.5	12 ± 3.1	18 ± 6.7	**p<0.05
	Fumaric acid	HMDB00134	2.9 ± 0.6	3.6 ± 0.6	16 ± 1.6	*p<0.001
	Malic acid	HMDB00156,HMDB00744	18 ± 2.9	24 ± 4.9	71 ± 1.5	*p<0.001
glyconeogenesis/glycogenolysis	Glucose 1-phosphate	HMDB01586	1.4 ± 0.1	2.1 ± 0.6	6.2 ± 1.0	*p<0.001
fatty acid synthesis	Malonyl CoA_divalent	HMDB01175	N.D.	N.D.	N.D.	
Triacylglycerol synthesis	Glycerol 3-phosphate	HMDB00126	34 ± 0.4	30 ± 7.9	99 ± 18	*p<0.01

Table S2 (continued)

Prine	Inosine monophosphate	HMDB00175	5.9 ± 1.8	4.9 ± 1.4	12 ± 1.0	*p<0.001
	Adenosine monophosphate	HMDB00045	20 ± 8	56 ± 55	200 ± 71	*p<0.05
	Adenosine diphosphate	HMDB01341	118 ± 25	163 ± 56	300 ± 23	*p<0.05
	Adenosine triphosphate	HMDB00538	306 ± 82	386 ± 145	897 ± 237	*p<0.05
	Cyclic adenosine 3',5'-phosphate	HMDB00058	0.9 ± 0.5	0.6 ± 0.1	1.2 ± 0.4	0.32
	Deoxyadenosine triphosphate	HMDB01532	N.D.	N.D.	N.D.	
	Guanosine monophosphate	HMDB01397	1.7 ± 0.2	2.9 ± 1.8	13 ± 4.7	*p<0.05
	Guanosine diphosphate	HMDB01201	5.8 ± 1.8	12 ± 7.8	26 ± 4.6	*p<0.05
	Guanosine triphosphate	HMDB01273	24 ± 10	37 ± 18	54 ± 15	0.12
	Cyclic 3',5'-guanosine monophosphate	HMDB01314	N.D.	N.D.	N.D.	
Pirimidine	Uridine monophosphate	HMDB00288	2.2 ± 0.5	4.4 ± 2.7	24 ±12	**p<0.05
	Uridine diphosphate	HMDB00295	4.8 ± 1.0	12 ± 11	44 ± 13	*p<0.05
	Uridine triphosphate	HMDB00285	30 ± 8.7	52 ± 20	73 ± 17	**p<0.05
	Cytidine triphosphate	HMDB00082	2.8 ± 0.8	3.7 ± 1.1	15 ± 5.5	*p<0.01
	Cytidine-diphosphate	HMDB01546	0.7 ± 0.1	1.8 ± 1.6	6.4 ± 1.7	*p<0.05
	Cytidine monophosphate	HMDB00095	0.6 ± 0.1	1.0 ± 0.7	5.2 ± 2.1	*p<0.05
	Deoxycytidine triphosphate	HMDB00998	N.D.	N.D.	N.D.	
	Deoxythymidine monophosphate	HMDB01227	N.D.	N.D.	N.D.	
	Deoxythymidine 5'-diphosphate	HMDB01274	N.D.	N.D.	N.D.	
	Deoxythymidine triphosphate	HMDB01342	N.D.	N.D.	N.D.	
others	2-Oxoisovaleric acid	HMDB00019	0.4 ± 0.1	0.5 ± 0.1	1.7 ± 0.2	*p<0.001
	Glycolic acid	HMDB00115	N.D.	N.D.	12 ± 5.2	*p<0.01
	Glyoxylic acid	HMDB00119	N.D.	N.D.	N.D.	
	3-Hydroxybutyric acid	HMDB00011,HMDB00357,H	4.2 ± 0.6	6.9 ± 0.8	24 ± 1.7	*p<0.001
	2-Hydroxybutyric acid	HMDB00008	N.D.	0.8 ± 0.3	1.6 ± 0.2	*p<0.05
	Nicotinamide adenine dinucleotide^{+}	HMDB00902	77 ± 17	105 ± 11	131 ± 5	**p<0.01
	Nicotinamide adenine dinucleotide phosphate^{+}	HMDB00217	2.0 ± 0.9	2.8 ± 0.8	8.0 ± 1.9	*p<0.01
	CoA_divalent	HMDB01423	N.D.	N.D.	N.D.	
	Gluconic acid	HMDB00625	44 ± 10	6.5 ± 1.5	14 ± 4.0	***p<0.01

* femoral artery with macrophage-rich neointima vs. normal femoral artery and femoral artery with SMC-rich neointima

** femoral artery with macrophage-rich neointima vs. normal femoral artery

*** normal femoral artery vs. femoral arteries with SMC-rich and macrophage-rich neointima

N.D.: not detected
