# Supplementary material for: Increased Metabolite Levels of Glycolysis and Pentose Phosphate Pathway in Rabbit Atherosclerotic Arteries and Hypoxic Macrophage
Source: PLoS One. 2014 Jan 23;9(1):e86426. doi: 10.1371/journal.pone.0086426 (PMC3900532; doi:10.1371/journal.pone.0086426)
Supplement: Table S3 — Quantities of macrophage metabolites. (PDF) [file pone.0086426.s003.pdf]

Table S3 Quantities of macrophage metabolites

Pathway	Metabolites	HMDB ID	Concentration (pmol/10^6cells) PMA, normoxia (control) n=6, mean ± SD	PMA+LPS+INFγ, normoxia (M1 normoxia) n=6, mean ± SD	PMA+LPS+INFγ, hypoxia (M1 hypoxia) n=6, mean ± SD	p value one-way ANOVA /Bonfferoni post test
Glycolysis	Glucose 6-phosphate	HMDB01401	3274 ± 247	1863 ± 163	6147 ± 351	*<0.001, **<0.001
	Fructose 6-phosphate	HMDB00124	833 ± 79	569 ± 33	1793 ± 71	*<0.001, **<0.001
	Fructose 1,6-diphosphate	HMDB01058	1034 ± 156	2113 ± 365	11770 ± 1253	**<0.001
	Dihydroxyacetone phosphate	HMDB01473	310 ± 71	385 ± 83	2346 ± 1395	**<0.01
	Glyceraldehyde 3-phosphate	HMDB01112	183 ± 17	289 ± 83	625 ± 267	**<0.01
	3-Phosphoglyceric acid	HMDB00807	434 ± 41	399 ± 47	611 ± 52	**<0.001
	2-Phosphoglyceric acid	HMDB03391	N.D.	N.D.	N.D.	
	Phosphoenolpyruvic acid	HMDB00263	222 ± 30	182 ± 35	245 ± 35	**<0.05
	Pyruvic acid	HMDB00243	N.D.	N.D.	N.D.	
	Lactic acid	HMDB00190,HMDB01311	74335 ± 3276	47872 ± 4622	115892 ± 9843	*<0.001, **<0.001
Pentose phosphate cycle	6-Phosphogluconic acid	HMDB01316	225 ± 29	136 ± 13	302 ± 32	*<0.001, **<0.001
	Ribulose 5-phosphate	HMDB00618	213 ± 35	177 ± 44	781 ± 175	**<0.001
	Ribose 5-phosphate	HMDB01548	100 ± 46	71 ± 36	246 ± 123	**<0.01
	Sedoheptulose 7-phosphate	HMDB01068	806 ± 70	939 ± 54	1803 ± 59	*<0.01, **<0.001
	Erythrose 4-phosphate	HMDB01321	N.D.	N.D.	N.D.	
	Phosphoribosyl pyrophosphate	HMDB00280	1527 ± 256	614 ± 74	759 ± 156	*<0.001
Tricarboxylic acid cycle	Acetyl CoA_divalent	HMDB01206	N.D.	N.D.	N.D.	
	Citric acid	HMDB00094	14770 ± 402	12605 ± 835	8712 ± 179	*<0.001, **<0.001
	cis-Aconitic acid	HMDB00072	889 ± 42	757 ± 50	600 ± 25	*<0.001, **<0.001
	Isocitric acid	HMDB00193	701 ± 45	516 ± 34	506 ± 14	*<0.001
	2-Oxoglutaric acid	HMDB00208	1789 ± 259	2046 ± 122	2540 ± 109	*<0.05, **<0.001
	Succinic acid	HMDB00254	822 ± 238	705 ± 149	543 ± 97	0.04
	Fumaric acid	HMDB00134	1869 ± 60	1327 ± 129	1331 ± 147	*<0.001
	Malic acid	HMDB00156,HMDB00744	7124 ± 107	5412 ± 420	5010 ± 203	*<0.001, **<0.05
glyconeogenesis/glycogenolysis	Glucose 1-phosphate	HMDB01586	558 ± 29	603 ± 72	1911 ± 75	**<0.001
fatty acid synthesis	Malonyl CoA_divalent	HMDB01175	N.D.	N.D.	N.D.	
Triacylglycerol synthesis	Glycerol 3-phosphate	HMDB00126	8161 ± 508	4348 ± 280	11827 ± 362	*<0.001, **<0.001

Table S3 (continued)

Prine	Adenine	HMDB00034	11 ± 2	9 ± 3	14 ± 4	0.54
	Adenosine	HMDB00050	344 ± 25	132 ± 41	126 ± 12	*<0.001
	Adenosine diphosphate	HMDB01341	5886 ± 335	3501 ± 322	2981 ± 265	*<0.001, **<0.05
	Adenosine monophosphate	HMDB00045	439 ± 52	369 ± 36	343 ± 47	*<0.05
	Adenosine triphosphate	HMDB00538	54954 ± 1458	43501 ± 2756	43320 ± 1384	**<0.001
	Cyclic 3',5'-guanosine monophosphate	HMDB01314	N.D.	N.D.	N.D.	
	Cyclic adenosine 3',5'-monophosphate	HMDB00058	N.D.	N.D.	N.D.	
	Deoxyadenosine triphosphate	HMDB01532	N.D.	N.D.	N.D.	
	Guanine	HMDB00132	N.D.	N.D.	N.D.	
	Guanosine	HMDB00133	18 ± 5	N.D.	10 ± 0.3	*<0.001
	Guanosine diphosphate	HMDB01201	908 ± 57	699 ± 63	763 ± 58	**<0.001
	Guanosine monophosphate	HMDB01397	122 ± 7	119 ± 13	144 ± 42	0.2
	Guanosine triphosphate	HMDB01273	10947 ± 351	8841 ± 707	12749 ± 481	*<0.001, **<0.001
	Inosine	HMDB00195	44 ± 39	N.D.	80 ± 26	*<0.05, **<0.001
	Inosine monophosphate	HMDB00175	240 ± 18	276 ± 56	2717 ± 747	**<0.001
	S-Adenosylmethionine	HMDB01185	526 ± 72	404 ± 31	394 ± 26	*<0.01
Pirimidine	Cytidine	HMDB00089	108 ± 5	50 ± 9	69 ± 13	*<0.001, **<0.01
	Cytidine diphosphate	HMDB01546	307 ± 24	203 ± 37	164 ± 18	*<0.001
	Cytidine monophosphate	HMDB00095	75 ± 6	57 ± 8	48 ± 3	0.0009
	Cytidine triphosphate	HMDB00082	6101 ± 168	4722 ± 391	5330 ± 383	*<0.001, **<0.05
	Cytosine	HMDB00630	N.D.	N.D.	N.D.	
	Deoxycytidine triphosphate	HMDB00998	N.D.	N.D.	N.D.	
	Deoxythymidine 5'-diphosphate	HMDB01274	N.D.	N.D.	N.D.	
	Deoxythymidine monophosphate	HMDB01227	N.D.	N.D.	N.D.	
	Deoxythymidine triphosphate	HMDB01342	N.D.	N.D.	N.D.	
	Hypoxanthine	HMDB00157	N.D.	N.D.	106 ± 87	0.11
	Thymidine	HMDB00273	N.D.	N.D.	N.D.	
	Thymine	HMDB00262	N.D.	N.D.	N.D.	
	Uracil	HMDB00300	N.D.	N.D.	N.D.	
	Uridine	HMDB00296	N.D.	N.D.	N.D.	
	Uridine diphosphate	HMDB00295	806 ± 139	343 ± 50	298 ± 42	*<0.001
	Uridine monophosphate	HMDB00288	135 ± 18	129 ± 10	94 ± 26	**<0.05
	Uridine triphosphate	HMDB00285	9172 ± 195	5138 ± 597	6229 ± 284	*<0.001, **<0.001

Table S3 (continued)

Amino acid	2-Hydroxybutyric acid	HMDB00008	N.D.	176 ± 21	261 ± 21	*<0.001, **<0.001
	2-Oxoisovaleric acid	HMDB00019	39 ± 12	N.D.	80	0.51
	3-Hydroxybutyric acid	HMDB00011,HMDB00357,H	180 ± 18	202 ± 36	272 ± 16	**<0.05
	Alanine	HMDB00161,HMDB01310	7319 ± 589	9388 ± 1081	13360 ± 515	*<0.001, **<0.001
	Anthranilic acid	HMDB01123	10 ± 1	17 ± 1	22 ± 7	*<0.05
	Arginine	HMDB00517,HMDB03416	6153 ± 255	6211 ± 978	8393 ± 705	**<0.001
	Aspartic acid	HMDB00191,HMDB06483	27771 ± 2195	21518 ± 1928	8156 ± 346	*<0.001, **<0.001
	Asparagine	HMDB00168	17579 ± 845	18864 ± 2521	22154 ± 927	**<0.01
	Betaine	HMDB00043	1135 ± 62	1137 ± 80	1143 ± 46	0.98
	Betaine aldehyde_+H_2O	HMDB01252	N.D.	N.D.	N.D.	
	Carnosine	HMDB00033	95 ± 6	52 ± 6	60 ± 3	*<0.001
	Choline	HMDB00097	1738 ± 163	4110 ± 353	7853 ± 887	*<0.001, **<0.001
	Citrulline	HMDB00904	42 ± 5	37 ± 6	60 ± 4	**<0.001
	Creatine	HMDB00064	6084 ± 371	6219 ± 586	6403 ± 447	0.52
	Creatinine	HMDB00562	252 ± 18	243 ± 22	251 ± 26	0.73
	Cysteine	HMDB00574,HMDB03417	411 ± 431	257 ± 83	64 ± 25	0.09
	Glutamic acid	HMDB00148,HMDB03339	224692 ± 5795	220431 ± 21017	224011 ± 11292	0.86
	Glutamine	HMDB00641,HMDB03423	18831 ± 3188	33488 ± 4303	53780 ± 3210	*<0.001, **<0.001
	Glycine	HMDB00123	56622 ± 1412	55635 ± 5831	63204 ± 3282	**<0.01
	Glycolic acid	HMDB00115	N.D.	N.D.	N.D.	
	Glyoxylic acid	HMDB00119	N.D.	N.D.	N.D.	
	Histidine	HMDB00177	2537 ± 162	2553 ± 310	3325 ± 147	**<0.001
	Homoserine	HMDB00719	N.D.	N.D.	N.D.	
	Hydroxyproline	HMDB00725	6933 ± 383	7054 ± 694	8775 ± 280	**<0.001
	Isoleucine	HMDB00172	7104 ± 308	7041 ± 965	9103 ± 258	**<0.001
	Leucine	HMDB00687	6178 ± 395	6473 ± 758	8390 ± 377	**<0.001
	Lysine	HMDB00182,HMDB03405	1349 ± 116	1538 ± 222	2224 ± 182	**<0.001
	Methionine	HMDB00696	1897 ± 143	1820 ± 207	2384 ± 128	**<0.001
	N,*N*-Dimethylglycine	HMDB00092	N.D.	N.D.	N.D.	
	Ornithine	HMDB00214,HMDB03374	295 ± 28	277 ± 48	421 ± 64	**<0.001
	Phenylalanine	HMDB00159	2195 ± 142	2118 ± 316	3039 ± 91	**<0.001

Table S3 (continued)

Amino acid	Proline	HMDB00162,HMDB03411	5375 ± 297	7250 ± 1030	8338 ± 436	*<0.001, **<0.05
	Putrescine	HMDB01414	49 ± 1	11	N.D.	*<0.001
	Sarcosine	HMDB00271	29 ± 6	N.D.	N.D.	*<0.001
	Serine	HMDB00187,HMDB03406	3305 ± 173	8652 ± 811	9791 ± 420	*<0.001, **<0.01
	Spermidine	HMDB01257	279 ± 52	213 ± 27	237 ± 32	*<0.05
	Spermine	HMDB01256	318 ± 118	246 ± 40	274 ± 34	0.27
	Threonine	HMDB00167	5252 ± 300	5783 ± 819	7623 ± 353	**<0.001
	Tryptophan	HMDB00929	48 ± 3	45 ± 8	53 ± 10	0.27
	Tyramine	HMDB00306	N.D.	N.D.	N.D.	
	Tyrosine	HMDB00158	2397 ± 154	2345 ± 323	3159 ± 112	**<0.001
	Valine	HMDB00883	3368 ± 158	3495 ± 510	4704 ± 198	**<0.001
	β-Alanine	HMDB00056	5353 ± 466	2773 ± 185	2322 ± 95	*<0.001, **<0.05
	γ-Aminobutyric acid	HMDB00112	222 ± 24	662 ± 97	889 ± 43	*<0.001, **<0.001
Other	CoA_divalent	HMDB01423	223 ± 150	252 ± 57	205 ± 113	0.77
	Gluconic acid	HMDB00625	563 ± 46	279 ± 32	358 ± 15	*<0.001, **<0.01
	Glutathione (GSH)	HMDB00125	71447 ± 58740	83748 ± 17663	49246 ± 27353	0.32
	Glutathione (GSSG)_divalent	HMDB03337	33149 ± 18559	18161 ± 12879	30432 ± 9962	0.19
	Nicotinamide adenine dinucleotide phosphate⁺	HMDB00217	614 ± 64	367 ± 97	435 ± 42	*<0.001
	Nicotinamide adenine dinucleotide⁺	HMDB00902	5797 ± 405	2890 ± 291	2661 ± 139	*<0.001

* control vs. M1 normoxia

** M1 normoxia vs. M1 hypoxia

N.D.: not detected

PMA: phorbol 12-myristate 13-acetate, LPS: lipopolysaccharide, INF-γ: interferon-γ
