# Supplementary material for: Increased Metabolite Levels of Glycolysis and Pentose Phosphate Pathway in Rabbit Atherosclerotic Arteries and Hypoxic Macrophage
Source: PLoS One. 2014 Jan 23;9(1):e86426. doi: 10.1371/journal.pone.0086426 (PMC3900532; doi:10.1371/journal.pone.0086426)
Supplement: Table S4 — Relation between plasminogen activator inhibitor-1 and metabolite levels. (PDF) [file pone.0086426.s004.pdf]

Table S4 Relation between plasminogen activator inhibitor−1 and metabolite levels

Pathway	Metabolites	HMDB ID	correlation cofficient	p value
Glycolysis	Glucose 6-phosphate	HMDB01401	−0.23	0.3611
	Fructose 6-phosphate	HMDB00124	−0.34	0.1731
	Fructose 1,6-diphosphate	HMDB01058	−0.59	<0.01
	Dihydroxyacetone phosphate	HMDB01473	−0.45	0.0634
	Glyceraldehyde 3-phosphate	HMDB01112	−0.56	<0.05
	3-Phosphoglyceric acid	HMDB00807	−0.37	0.1282
	Phosphoenolpyruvic acid	HMDB00263	0.09	0.715
	Lactic acid	HMDB00190,HMDB01311	−0.16	0.5268
Pentose phosphate cycle	6-Phosphogluconic acid	HMDB01316	0.00	0.9957
	Ribulose 5-phosphate	HMDB00618	−0.45	0.0589
	Ribose 5-phosphate	HMDB01548	−0.25	0.3202
	Sedoheptulose 7-phosphate	HMDB01068	−0.62	<0.01
	Phosphoribosyl pyrophosphate	HMDB00280	0.86	<0.0001
Tricarboxylic acid cycle	Citric acid	HMDB00094	0.77	<0.001
	cis-Aconitic acid	HMDB00072	0.81	<0.0001
	Isocitric acid	HMDB00193	0.93	<0.0001
	2-Oxoglutaric acid	HMDB00208	−0.72	<0.001
	Succinic acid	HMDB00254	0.53	<0.05
	Fumaric acid	HMDB00134	0.91	<0.0001
	Malic acid	HMDB00156,HMDB00744	0.94	<0.0001
glyconeogenesis/glycogenolysis	Glucose 1-phosphate	HMDB01586	−0.55	<0.05
Triacylglycerol synthesis	Glycerol 3-phosphate	HMDB00126	−0.03	0.901

Table S4 (continued)

Prine	Adenine	HMDB00034	0.03	0.9056
	Adenosine	HMDB00050	0.95	<0.0001
	Adenosine diphosphate	HMDB01341	0.95	<0.0001
	Adenosine monophosphate	HMDB00045	0.67	<0.01
	Adenosine triphosphate	HMDB00538	0.93	<0.0001
	Guanosine	HMDB00133	0.87	<0.0001
	Guanosine diphosphate	HMDB01201	0.79	<0.0001
	Guanosine monophosphate	HMDB01397	−0.14	0.5702
	Guanosine triphosphate	HMDB01273	0.00	0.9876
	Inosine	HMDB00195	−0.01	0.9656
	Inosine monophosphate	HMDB00175	−0.51	<0.05
	S-Adenosylmethionine	HMDB01185	0.78	0.0001
Pirimidine	Cytidine	HMDB00089	0.88	<0.0001
	Cytidine diphosphate	HMDB01546	0.87	<0.0001
	Cytidine monophosphate	HMDB00095	0.70	<0.01
	Cytidine triphosphate	HMDB00082	0.75	<0.001
	Hypoxanthine	HMDB00157	−0.26	0.2918
	Uridine diphosphate	HMDB00295	0.91	<0.0001
	Uridine monophosphate	HMDB00288	0.48	<0.05
	Uridine triphosphate	HMDB00285	0.91	<0.0001

Table S4 (continued)

Amino acid	2-Oxoisovaleric acid	HMDB00019	0.10	0.6984
	3-Hydroxybutyric acid	HMDB00011,HMDB00357,H	−0.60	<0.01
	Alanine	HMDB00161,HMDB01310	−0.74	<0.001
	Anthranilic acid	HMDB01123	−0.70	<0.01
	Arginine	HMDB00517,HMDB03416	−0.47	0.051
	Aspartic acid	HMDB00191,HMDB06483	0.74	<0.001
	Asparagine	HMDB00168	−0.59	<0.05
	Betaine	HMDB00043	−0.06	0.822
	Carnosine	HMDB00033	0.92	<0.0001
	Choline	HMDB00097	−0.79	0.0001
	Citrulline	HMDB00904	−0.32	0.1895
	Creatine	HMDB00064	−0.27	0.2822
	Creatinine	HMDB00562	0.15	0.565
	Cysteine	HMDB00574,HMDB03417	0.40	0.0994
	Glutamic acid	HMDB00148,HMDB03339	0.07	0.7916
	Glutamine	HMDB00641,HMDB03423	−0.81	<0.0001
	Glycine	HMDB00123	−0.29	0.24
	Histidine	HMDB00177	−0.48	<0.05
	Hydroxyproline	HMDB00725	−0.52	<0.05
	Isoleucine	HMDB00172	−0.44	0.071
	Leucine	HMDB00687	−0.56	<0.05
	Lysine	HMDB00182,HMDB03405	−0.63	<0.01
	Methionine	HMDB00696	−0.37	0.136
	Ornithine	HMDB00214,HMDB03374	−0.33	0.1865
	Phenylalanine	HMDB00159	−0.44	0.0698

Table S4 (continued)

Amino acid	Proline	HMDB00162,HMDB03411	−0.84	<0.0001
	Putrescine	HMDB01414	0.97	<0.0001
	Sarcosine	HMDB00271	0.96	<0.0001
	Serine	HMDB00187,HMDB03406	−0.96	<0.0001
	Spermidine	HMDB01257	0.48	<0.05
	Spermine	HMDB01256	0.29	0.2435
	Threonine	HMDB00167	−0.63	<0.01
	Tryptophan	HMDB00929	−0.02	0.9439
	Tyrosine	HMDB00158	−0.44	0.0701
	Valine	HMDB00883	−0.54	<0.05
	β-Alanine	HMDB00056	0.95	<0.0001
	γ-Aminobutyric acid	HMDB00112	−0.92	<0.0001
Other	CoA_divalent	HMDB01423	−0.07	0.7777
	Gluconic acid	HMDB00625	0.90	<0.0001
	Glutathione (GSH)	HMDB00125	0.03	0.9155
	Glutathione (GSSG)_divalent	HMDB03337	0.30	0.2189
	Nicotinamide adenine dinucleotide phosphate^{+}	HMDB00217	0.97	<0.0001
	Nicotinamide adenine dinucleotide^{+}	HMDB00902	0.80	<0.0001
